# Comment on Chen, J.; Su, Y.; Si, H.; Chen, J. Managerial Areas of Construction and Demolition Waste: A Scientometric Review. *Int. J. Environ. Res. Public Health* 2018, *15*, 2350

**DOI:** 10.3390/ijerph16101837

**Published:** 2019-05-23

**Authors:** Yuh-Shan Ho

**Affiliations:** Trend Research Centre, Asia University, No. 500, Lioufeng Road, Wufeng, Taichung County 41354, Taiwan; ysho@asia.edu.tw; Tel.: +886-4-2332-3456 (ext. 1797); Fax: +886-4-2330-583

“Managerial areas of construction and demolition waste: A scientometric review” by Chen et al. was recently published in the journal [1]. Most of the related results mentioned in the original paper [1] are unacceptable because of the use of inappropriate search filters. Chen et al. stated in Section 2.1. Data Collection that ‘Data for the contribution was extracted from the WoS Core collection database (SCI-EXPANDED, SSCI) in May 2018.’ and ‘The final search terms included “TS = (“construction waste *” OR “demolition waste *” OR “construction and demolition waste *” OR “CDW” OR “C&DW”) AND TS = (“management” OR “managerial” OR “managing” OR “manage”)”. The language of the publications was limited to English and document type was limited to articles, and the time span was set to 1975–2018. As a result, 398 bibliographic records were retrieved.’

Searching keywords used in the original paper [1] were inappropriate. “CDW” was used as a searching keyword, however, many of the publications were irrelevant “construction and demolition waste”, for instance, cell dry weight (CDW), community disability workers (CDW), Cada Dia Welsh (CDW), cellular dry weight (CDW), collaborative design workshop (CDW), cotton direct-seeded after wheat (CDW), clinical data warehouse (CDW), corrected dry weights (CDW), clipping dry weight (CDW), Changjiang diluted water (CDW), corporate data warehouse (CDW), coarse dead wood (CDW), central data warehouse (CDW), conventional diagnostic work-up (CDW), colonial development and welfare (CDW), circumpolar deep water (CDW), and CPP Ile-de-France II-CDW_ 2016_0014.

A better way to improve this method would be to search data from SCI-EXPANDED and SSCI (updated on 25 March 2019) by using (“construction waste” or “construction wastes” or “demolition waste” or “demolition wastes” or “C&DW” or “C&D waste”) and (“management” or “manager” or “managers” or “manage” or “managing” or “managed” or “manageable”) as keywords in terms of the topic (including title, abstract, author keywords, and *KeyWords Plus*) within the publication years between 1975–2018. The limitation was set to the language being only English and the document type being only articles. This method resulted in 496 articles (125% of 398 articles). From the original paper [1], the authors stated that “document type was limited to articles”, however, two reviews “Trend of the research on construction and demolition waste management” [2] and “Quantifying construction and demolition waste: an analytical review” [3] were found in Table 2.

The Web of Science Core Collection is designed for researchers to find published literature, and not for bibliometric studies [4,5]. Therefore, using the Web of Science Core Collection with an accurate bibliometric method is critical for all researchers [4,5]. It was pointed out that the documents searched out by *KeyWords Plus* were irrelevant to “construction and demolition waste” [6]. Due to biases from the Web of Science Core Collection, Ho’s group was the first to propose “front page” (including the article title, the abstract, and the author keywords) as a filter to improve the bibliometric method [7,8,9]. Furthermore, a more accurate bibliometric method was applied with “front page” as a filter. In all, 2591 documents (80% of the 3226 documents) were found including 369 articles (74% of 496 articles) by searching keywords in their “front page” while 127 articles (26% of 496 articles) were likely to be irrelevant to “construction and demolition waste”, for example, articles entitled “Removal of cement mortar remains from recycled aggregate using pre-soaking approaches” [10], “Dynamic material flow analysis for Norway’s dwelling stock” [11], and “Intrapleural hyperthermic perfusion using distilled water at 48 °C for malignant pleural effusion” [12]. It is clear that utilizing the “front page” as a filter can avoid introducing unrelated articles for analysis [7,13]. In recent years, similar rebuttals have also been published in *Environmental Science and Pollution Research* [4] and *Renewable & Sustainable Energy Reviews* [5].

Furthermore, results by using the “front page” field resulted in 11 highly cited articles with 100 or more total citations from We of Science Core Collection (*TC*_2018_ ≥ 100) [14], as shown in Table 1.

The citation indicator, *TC*_2018_, the total number of citations from Web of Science Core Collection since publication to the end of 2018 [26,27] was also presented in Table 1. The advantage of *TC*_2018_ is that they are invariable and ensure repeatability in comparison to the index of citation from the Web of Science [7]. The data were collected on 25 March in 2019. A bias might be obtained as some publications in 2018 have not yet been updated in the Web of Science Core Collection.

Chen et al. used inappropriate searching keywords and methods to publish a bibliometric article in the *International Journal of Environmental Research and Public Health*, which may result in misleading journal readers. From my perspective, Chen et al. could have provided a more accurate result if they had used appropriate searching keywords and method. In addition, using such a limited number of papers for a scientometric review is inappropriate from a statistical point of view.

## Figures and Tables

**Table 1:** Top 11 highly cited articles.

Authors	Article Title	Year	Journals	*TC*_2018_
Barros et al.	A two-level network for recycling sand: A case study [15]	1998	European Journal of Operational Research	247
Rao et al.	Use of aggregates from recycled construction and demolition waste in concrete [16]	2007	Resources Conservation and Recycling	231
Spengler et al.	Environmental integrated introduction and recycling management [17]	1997	European Journal of Operational Research	192
Bossink and Brouwers	Construction waste: Quantification and source evaluation [18]	2001	Journal of Construction Engineering and Management	176
Poon et al.	On-site sorting of construction and demolition waste in Hong Kong [19]	2001	Resources Conservation and Recycling	163
Jaillon et al.	Quantifying the waste reduction potential of using prefabrication in building construction in Hong Kong [20]	2009	Waste Management	136
Osmani et al.	Architects’ perspectives on construction waste reduction by design [21]	2008	Waste Management	123
Kartam et al.	Environmental management of construction and demolition waste in Kuwait [22]	2004	Waste Management	105
Bertram et al.	The contemporary European copper cycle: waste management subsystem [23]	2002	Ecological Economics	102
Ekanayake and Ofori	Building waste assessment score: design-based tool [24]	2004	Building and Environment	101
Jim	Urban soil characteristics and limitations for landscape planting in Hong Kong [25]	1998	Landscape and Urban Planning	101

*TC*_2018_: number of citations from the Web of Science Core Collection since publication to the end of 2018.
